# *Bacillus subtilis* 5′-nucleotidases with various functions and substrate specificities

**DOI:** 10.1186/s12866-016-0866-5

**Published:** 2016-10-26

**Authors:** Ayako Terakawa, Ayane Natsume, Atsushi Okada, Shogo Nishihata, Junko Kuse, Kosei Tanaka, Shinji Takenaka, Shu Ishikawa, Ken-ichi Yoshida

**Affiliations:** 1 Department of Agrobioscience, Kobe University, 1-1 Rokkodai, Nada, Kobe, Hyogo 657-8501 Japan; 2 Organization of Advanced Science and Technology, Kobe University, 1-1 Rokkodai, Nada, Kobe, Hyogo 657-8501 Japan; 3 Department of Science, Technology and Innovation, Kobe University, 1-1 Rokkodai, Nada, Kobe, Hyogo 657-8501 Japan

**Keywords:** *Bacillus subtilis*, Haloacid dehalogenase superfamily, Inositol monophosphatase, Inositol phosphate, Nucleoside/nucleotide metabolism, 5′-nucleotidase, Oxidative stress, Phosphatase, Protein motif

## Abstract

**Background:**

In *Escherichia coli*, *nagD*, *yrfG*, *yjjG*, *yieH*, *yigL*, *surE*, and *yfbR* encode 5′-nucleotidases that hydrolyze the phosphate group of 5′-nucleotides. In *Bacillus subtilis*, genes encoding 5′-nucleotidase have remained to be identified.

**Results:**

We found that *B. subtilis ycsE, araL*, *yutF*, *ysaA*, and *yqeG* show suggestive similarities to *nagD*. Here, we expressed them in *E. coli* to purify the respective His_6_-tagged proteins. YcsE exhibited significant 5′-nucleotidase activity with a broader specificity, whereas the other four enzymes had rather weak but suggestive activities with various capacities and substrate specificities. In contrast, *B. subtilis yktC* shares high similarity with *E. coli suhB* encoding an inositol monophosphatase. YktC exhibited inositol monophosphatase activity as well as 5′-nucleotidase activity preferential for GMP and IMP. The *ycsE*, *yktC*, and *yqeG* genes are induced by oxidative stress and were dispensable, although *yqeG* was required to maintain normal growth on solid medium. In the presence of diamide, only mutants lacking *yktC* exhibited enhanced growth defects, whereas the other mutants without *ycsE* or *yqeG* did not.

**Conclusions:**

Accordingly, in *B. subtilis*, at least YcsE and YktC acted as major 5′-nucleotidases and the four minor enzymes might function when the intracellular concentrations of substrates are sufficiently high. In addition, YktC is involved in resistance to oxidative stress caused by diamide, while YqeG is necessary for normal colony formation on solid medium.

**Electronic supplementary material:**

The online version of this article (doi:10.1186/s12866-016-0866-5) contains supplementary material, which is available to authorized users.

## Background

The pool sizes of nucleotides and nucleosides are balanced to enable the efficient synthesis of DNA and RNA [1]. Numerous enzymes involved in the biosynthesis and catabolism of nucleic acids are controlled to regulate the appropriate pool size of each compound. For example, 5′-nucleotidases hydrolyze 5′-nucleotides to generate nucleosides and inorganic phosphate, which participate in the regulatory mechanism that opposes the generation of nucleotides with the phosphorylation of nucleosides catalyzed by kinases.

5′-Nucleotidases are ubiquitous among species and reside in different subcellular locations. Extracellular 5′-nucleotidases are produced by certain bacteria. For example, *Vibrio parahaemolyticus* NutA is a 5′-nucleotidase bound to the membrane by a lipid anchor [2]. *Escherichia coli* UshA is a periplasmic enzyme with 5′-nucleotidase and UDP-sugar hydrolase activities [3]. These 5′-nucleotidases degrade extracellular nucleotides to satisfy the cell’s nutritional requirements.

In contrast, bacteria produce numerous intracellular 5′-nucleotidases that belong to various enzyme families. In *E. coli*, the substrates of NagD are UMP, GMP, AMP, and CMP [4]. NagD belongs to the haloacid dehalogenase superfamily (HADSF) characterized by a specific protein motif [5]. The HADSF family comprises numerous proteins in organisms ranging from prokaryotes to higher eukaryotes, including humans. The vast majority of enzymes of the HADSF family are phosphoryl transferases, although the superfamily was named after 2-haloacid dehalogenase, because it is the first structurally characterized member. When the amino acid sequences of their entire coding regions are compared, similarities among the HADSF members are not usually very high (15–30 % identical), although their central regions involved in catalytic activity are relatively conserved. Further, correlation between structure and catalytic activity is frequently observed and interrelates with similarities among the structures of substrates [6–8]. *E. coli* produces other HADSF-family enzymes such as YrfG, YjjG, YieH, and YigL, and each exhibits 5′-nucleotide phosphatase activity [9, 10].

In addition, *E. coli* produces SurE and YfbR, which exhibit 5′-nucleotidase activity and do not belong to the HADSF family [10]. SurE shows broad substrate specificity, by dephosphorylating 5′-nucleotides as well as 3′-nucleotides, with highest affinity for 3′-AMP. SurE hydrolyzes polyphosphate with preference for short-chain substrates. Homologs of *E. coli surE* are present in numerous eubacteria and archaea, and SurE represents a family of metal-dependent phosphatases [11]. YfbR belongs to the HD domain superfamily of metal-dependent phosphatases and phosphodiesterases, and the HD domain was named after a protein motif with predicted catalytic residues containing the conserved doublet His–Asp [12]. YfbR specifically hydrolyzes 5′-deoxyribonucleotides.

Inositol monophosphatase denotes phosphatases that liberate inorganic phosphate from *myo*-inositol 1-monophosphate (MIMP). The mammalian and plant enzymes are involved in the metabolism of inositol phospholipids, including the generation and degradation of inositol phosphates and phosphatidylinositols that mediate cellular signal transduction [13]. Inositol monophosphatases are present in diverse organisms such as bacteria and higher eukaryotes, and evolved from a common ancestral gene [14, 15]. Mammalian inositol monophosphatases exhibit relatively broad substrate specificity for phosphate-containing compounds [16, 17]. For example, the enzyme isolated from rat testis hydrolyzes adenosine 2′-monophosphate [16] and the bovine brain enzyme hydrolyzes β-glycerophosphate and adenosine 2′-monophosphate [17]. *E. coli suhB* encodes a protein homologous to eukaryotic inositol monophosphatases with equivalent activities [18].

However, phosphatidylinositol is not present in *E. coli* usually [19], and its physiological function in cells is unknown. In contrast, *E. coli* SuhB exhibits wider substrate specificity and hydrolyzes β-glycerophosphate and adenosine 2′-monophosphate as well [18]. Thus, we hypothesized that bacterial inositol monophosphatases might also function as 5′-nucleotidase. Moreover, various *E. coli* enzymes likely dephosphorylate 5′-nucleotides with different specificities and functions and may therefore act together to maintain the sizes of the intracellular pools of nucleotides and nucleosides.

In *Bacillus subtilis*, the partial nucleotide limitation induces 5′-nucleotidase activity [20], and *B. subtilis* is used for fermentative inosine production, which may involve the dephosphorylation of IMP catalyzed by an unidentified 5′-nucleotidase [21]. However, *B. subtilis* genes encoding 5′-nucleotidase have remained to be identified.

Here we selected *B. subtilis* genes potentially encoding 5′-nucleotidases [22] and expressed them in *E. coli* to characterize their function. We identified two major and four minor genes encoding 5′-nucleotidases, with various functions and substrate specificities. A major 5′-nucleotidase was involved in resistance to oxidative stress, and a minor enzyme was required for normal growth on solid medium.

## Results

### Selection of *B. subtilis* genes similar to *E. coli* genes encoding 5′-nucleotidase

**Fig. 1:**
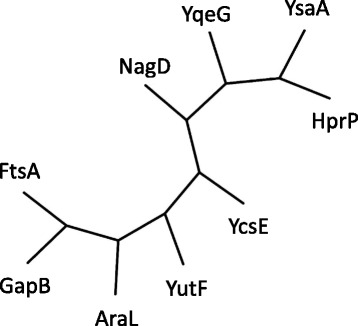
Similarities among the amino acid sequences of the proteins encoded by *E. coli nagD* homologs in the *B. subtilis* genome. The amino acid sequences of the proteins encoded by *B. subtilis araL*, *yutF*, *yqeG*, *ysaA*, *ycsE*, *gapB*, *ftsA*, and *hprP*, and *E. coli nagD* were analyzed using CLUSTALW (default fast/approximate settings [http://www.genome.jp/tools/clustalw/]) to generate the phylogenic tree

In *E. coli*, at least *nagD*, *yrfG*, *yjjG*, *yieH*, *yigL*, *surE*, and *yfbR* encode 5′-nucleotidases, and their gene products are classified in the families as follows: HADSF (NagD, YrfG, YjjG, YieH, and YigL), SurE, and HD domain (YfbR). In the *B. subtilis* genome, no gene shares significant homology with *surE* or *yfbR*. However, gene products of *araL*, *yutF*, *yqeG*, *ysaA*, *ycsE*, *gapB*, *ftsA*, and *hprP* share some suggestive similarities with that of *E. coli nagD* (Fig. 1); AraL (65.8 % similarity/272 aa overlap), YutF (73.0 %/256 aa), YqeG (15.7 %/172 aa), YsaA (16.9 %/260 aa), YcsE (51.4 %/249 aa), GapB (6.8 %/340 aa), FtsA (9.5 %/440 aa), and HprP (15.7 %/216 aa). Among them, AraL, YutF, YqeG, YsaA, YcsE, and HprP were supposed to belong to HADSF, since their amino acid sequences contain the HADSF motif (http://pfam.xfam.org/family/PF00702.24).

The *araL* gene resides within the L-arabinose operon, which encodes enzymes required for the degradation of L-arabinose and is under the control of AraR repressor to be induced in the presence of L-arabinose [23]. AraL was predicted to be a phosphatase that hydrolyzes certain sugar-phosphates [23].

The *yutF* gene encodes a protein that was predicted to be involved in N-acetyl-glucosamine catabolism and similar to 4-nitrophenyl phosphatase [24]. The condition-dependent transcriptome analysis revealed that *yutF* was almost constitutively expressed at low levels [25].

The *yqeG*, *ysaA*, and *ycsE* genes encode a putative HADSF-family enzyme of unknown function. The *yqeG* gene is the first gene of the long operon comprising *yqeG*, *yqeH*, *aroD*, *yqeI*, *nadD*, *yqeK*, *yqeL*, and *yqeM*. This operon encodes essential genes [26], which are expressed constitutively at significant levels that increase during germination and under conditions of oxidative stress [25]. The *ysaA* and *ycsE* genes are constitutively expressed and are induced, respectively, during the nutritional shift from malate to malate plus glucose and in the presence of ethanol or diamide [25].

The products of *gapB*, *ftsA*, and *hprP* show relatively lower similarities to NagD (Fig. 1). GapB is NADP-dependent glyceraldehyde-3-phosphate dehydrogenase, which is involved in gluconeogenesis [27]. FtsA is an actin-like ATPase involved in cell division [28]. And HprP is a P-Ser-HPr phosphatase involved in carbon catabolite repression [29].

### 5′-Nucleotidase activity of *B. subtilis* enzymes sharing some similarities with NagD

Of the eight *B. subtilis* genes that share some similarities with *nagD* as described above, *gapB*, *ftsA*, and *hprP* have respective and specific functions other than as 5′-nucleotidases [27–29]. Therefore, these genes were excluded from the present study. We cloned and expressed *araL*, *yutF*, *yqeG*, *ysaA*, and *ycsE* in *E. coli* as His_6_-tag fusion proteins.

**Fig. 2:**
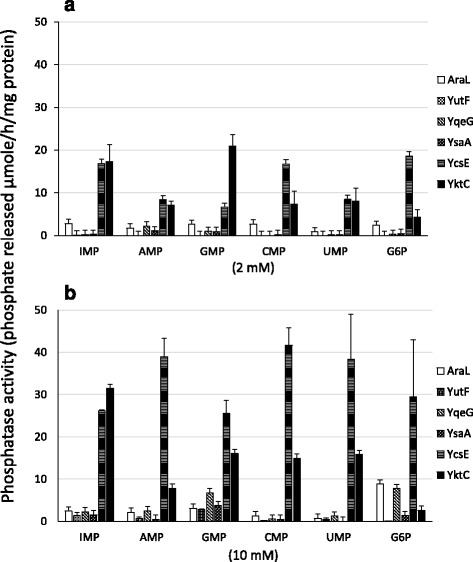
Phosphatase activities of *B. subtilis* proteins homologous to *E. coli* NagD. Each purified protein (0.25 mg/ml, indicated on the right) was incubated with 2 mM (**a**) or 10 mM (**b**) of various substrates for 12 h and assayed for phosphatase activity as described in Methods. All presented data are the mean values of three independent experiments ± SD

The His_6_-tag fusion proteins were purified to form respective single bands in sodium dodecyl sulfate polyacrylamide gel electrophoresis (SDS-PAGE, data not shown), and were subjected to phosphatase assays using the substrates as follows: AMP, CMP, GMP, IMP, UMP, and glucose 6-phosphate (G6P) (Fig. 2). G6P was included because it is the substrate of certain HADSF enzymes [9]. YcsE appeared to be the most efficient phosphatase acting on all six substrates at a lower concentration (2 mM), indicating its broad substrate specificity with preference for IMP, CMP, and G6P. Its activity increased in the presence of 10 mM substrates, particularly AMP, GMP, and UMP. The other four enzymes were relatively less active even at the higher substrate concentration. All 5′-nucleotides served almost equally as substrates for AraL at lower and higher concentrations, and AraL activity was enhanced particularly against G6P at the higher concentration. YutF exhibited purine 5′-nucleotidase activity with the substrates GMP and IMP, but only at the higher concentration, and did not hydrolyze pyrimidine nucleotides and G6P. YqeG hydrolyzed GMP and G6P only at the higher concentration. YsaA activity was weakest with purine-nucleotide substrates at the higher concentration.

The catalytic properties of YcsE are shown in Table 1. The substrate specificity of YcsE was broad with higher *K*_M_ values, suggesting that it efficiently dephosphorylated these substrates at higher concentrations. In contrast, the properties of the other four enzymes were not determined because of their lower activities. Nevertheless, the data do not exclude their physiological function as 5′-nucleotidases, because the activities of two of the 5′-nucleotidases YieH and YigL identified previously in *E. coli* were too low to determine their enzymatic properties [9].

**Table 1:** Kinetic constants of YcsE

Substrate	*K*_M_ (mM)^a^	*V*_*max*_ (μmol min^−1^ mg protein^−1^)^a^	*k*_*cat*_ (s^−1^)^a^
IMP	3.2 ± 0.036	0.13 ± 0.060	3.7 ± 1.4 × 10^2^
AMP	8.6 ± 0.80	0.50 ± 0.029	2.3 ± 0.13 × 10^3^
GMP	10.3 ± 0.12	0.45 ± 0.12	2.0 ± 0.55 × 10^3^
CMP	4.7 ± 0.23	0.53 ± 0.18	3.4 ± 0.011 × 10^3^
UMP	8.5 ± 0.69	0.20 ± 0.072	9.6 ± 3.5 × 10^2^
G6P	22.2 ± 0.96	2.3 ± 0.48	1.06 ± 0.22 × 10^4^

^a^All presented data are the mean values of three independent experiments ± SD

### 5′-Nucleotidase activity of SuhB homolog in *B. subtilis*

As described above, *E. coli* SuhB is an inositol monophosphatase, which exhibits wider substrate specificity, as it hydrolyzes β-glycerophosphate and adenosine 2′-monophosphate as well [18]. Thus, we hypothesized that bacterial inositol monophosphatases might also function as 5′-nucleotidase.

The *B. subtilis yktC* gene, which is the homolog of *E. coli suhB* (31.7 % similarity of 265 overlapping amino acid residues), was cloned and expressed as a His_6_-tagged protein in *E. coli*. His_6_-tagged YktC was purified to form a single band in SDS-PAGE (data not shown) and was subjected to phosphatase assays in the presence of various substrates (Fig. 2, Table 2). The inositol monophosphatase activity of YktC was indeed demonstrated by its efficient ability to hydrolyze MIMP, and it also turned out to exhibit 5′-nucleotidase activity preferentially against IMP and GMP as substrates. To our knowledge, this is the first identification of an inositol monophosphatase with 5′-nucleotidase activity. Compared with *E. coli* SuhB, YktC hydrolyzed G6P less efficiently, while β-glycerophosphate more efficiently [18]. Expression of *yktC* is mainly constitutive and is markedly enhanced by stressors such as diamide, ethanol, or high salt as well as at higher and lower temperatures [25].

**Table 2:** Kinetic constants of YktC

Substrate	*K*_M_ (mM)^a^	*V*_*max*_ (μmol min^−1^ mg protein^−1^)^a^	*k*_*cat*_ (s^−1^)^a^
MIMP	0.076 ± 0.006	0.82 ± 0.16	2.9 ± 0.33 × 10^3^
IMP	1.1 ± 0.083	0.078 ± 0.0084	1.8 ± 0.59 × 10^2^
AMP	1.8 ± 0.13	0.030 ± 0.00022	5.3 ± 0.039 × 10^1^
GMP	1.9 ± 0.026	0.169 ± 0.028	3.0 ± 0.34 × 10^2^
CMP	1.6 ± 0.017	0.049 ± 0.014	1.5 ± 0.52 × 10^2^
UMP	2.4 ± 0.052	0.11 ± 0.0099	9.8 ± 4.6 × 10^2^
G6P	2.6 ± 0.51	0.021 ± 0.010	3.7 ± 1.3 × 10^1^
β-glycerophosphate	0.49 ± 0.035	1.2 ± 0.32	5.7 ± 1.2 × 10^2^

^a^All presented data are the mean values of three independent experiments ± SD

### Physiological functions of YqeG, YcsE, and YktC

As mentioned above, *yqeG* expression is enhanced by oxidative stress, *ycsE* is induced in the presence of diamide or ethanol, and *yktC* is markedly enhanced by stressors such as diamide, ethanol, or high salt, as well as at higher and lower temperatures [25]. These findings inspired us to investigate the three genes encoding 5′-nucleotidase for their possible functional association with the response to oxidative stress.

**Fig. 3:**
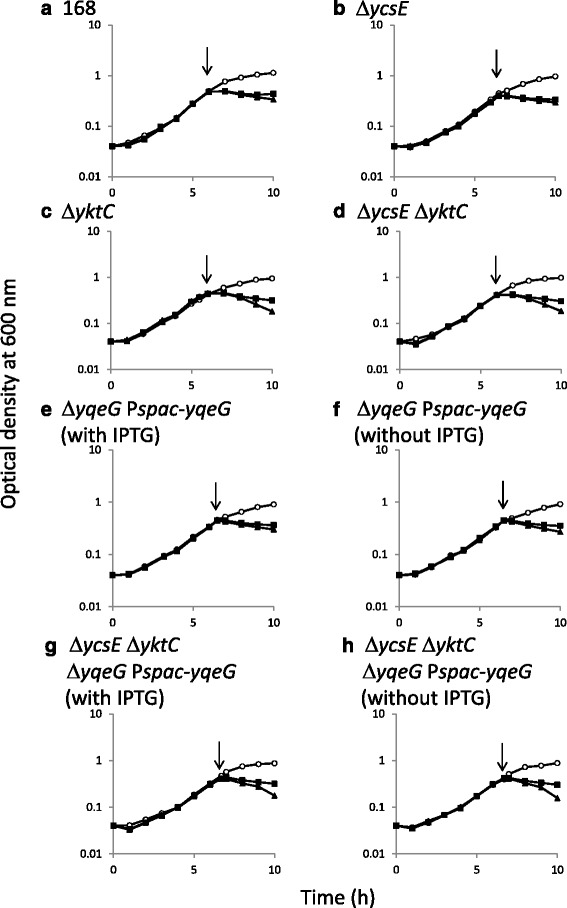
Growth curves of *B. subtilis* strains. Strains 168 (**a**), NON01 (**b** Δ*ycsE*), NON02 (**c** Δ*yktC*), NON03 (**d** Δ*ycsE* Δ*yktC*), NON05 (**e** and **f**, Δ*yqeG* P*spac-yqeG*), and NON06 (**g** and **h**, Δ*ycsE* Δ*yktC* Δ*yqeG* P*spac-yqeG*) were inoculated into liquid medium and their growth was monitored. At the times indicated by the arrowheads, diamide was added to final concentrations of 0 mM (open circle), 1 mM (solid square), and 4 mM (solid triangle). Strains NON05 and NON06 were grown in the presence (**e** and **g**) and absence (**f** and **h**) of 1 mM IPTG. All experiments were repeated more than three times and similar results were observed

**Fig. 4:**
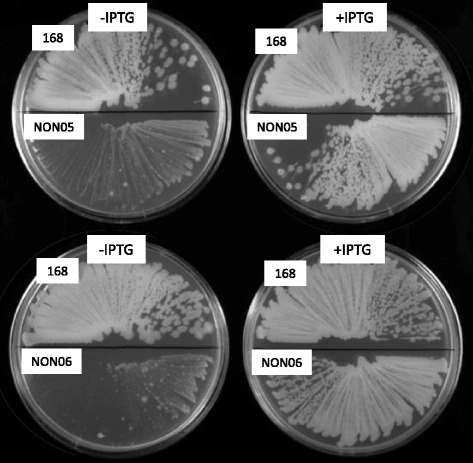
Colony formation by *B. subtilis* strains. Suspensions of strains 168, NON05, and NON06 were streaked on LB plates with (right) or without (left) 1 mM IPTG and incubated overnight at 37 °C. All experiments were repeated more than three times and similar results were observed

We used conventional marker replacement to inactivate *ycsE* and *yktC*. The *yqeG* gene is the first of the long operon comprising *yqeG*, *yqeH*, *aroD*, *yqeI*, *nadD*, *yqeK*, *yqeL*, and *yqeM* [26]. At least *yqeH*, *yqeI*, and *nadD* are essential genes [26]. Therefore, we tried to introduce an in-frame deletion of *yqeG* to maintain the expression of the downstream essential genes. However, numerous attempts failed. Therefore, once an additional copy of *yqeG* was introduced into the *amyE* locus under the control of *spac* promoter, which is conditionally induced by IPTG. And then, in the presence of IPTG, the existing *yqeG* gene was deleted using marker replacement so that the genes downstream were constitutively expressed by read-through from the kanamycin-resistance cassette (strain NON05). Regardless of the presence and absence of IPTG, strain NON05 grew in liquid medium (Fig. 3), suggesting that *yqeG* might be dispensable in planktonic growth. However, the *spac* promoter is somewhat leaky; a significant basal level of expression still exists in the absence of IPTG [30]. Therefore, in the absence of IPTG, there might be some production of YqeG, and this might be sufficient for planktonic growth. Nevertheless, NON05 formed fewer colonies in the absence of IPTG (Fig. 4), suggesting that *yqeG* was required to maintain normal growth particularly on solid medium. Similarly, less efficient colony-formation was exhibited by the triple mutant NON06 in the absence of IPTG, and colony formation was slightly decreased when *ycsE* and *yktC* were inactivated additionally.

The addition of diamide to liquid medium arrested the growth of the parental strain 168 (Fig. 3). The growth characteristics of the mutant NON01 lacking *ycsE* were comparable to those of the parental strain in the presence of diamide. Conversely, the mutants NON02 and NON03 lacking *yktC* exhibited the enhanced growth defect in the presence of the higher concentration of diamide (4 mM), as the optical density for cells decreased significantly possibly because of cell lysis. The results suggest that *yktC*, but not *ycsE*, contributed to resistance to oxidative stress induced by diamide. In contrast, NON05 did not exhibit significantly increased sensitivity to diamide, depending on the repression of *yqeG* in the absence of IPTG (Fig. 3). The triple-mutant NON06 exhibited the growth defect similar to that of NON02 or NON03 in the presence of diamide (Fig. 3), and the growth of NON06 did not depend on IPTG. Therefore, the conditional expression of *yqeG* was not associated with the susceptibility to diamide. Taken together, the data indicate that only YktC was involved in the resistance to oxidative stress.

## Discussion

In eukaryotes, multiple nucleotidases and nucleoside kinases control the intracellular concentration of nucleotides [31–33]. Nucleotidases play a role in the maintenance of a constant nucleoside supply, which is required to maintain RNA metabolism and biosynthesis of cell wall [1]. In bacteria, numerous nucleotidases with different substrate specificities control the intracellular nucleotide pool [34], and it is believed that the accumulation of intracellular nucleotides is potentially toxic and therefore must be prevented [31–33].

Here we show that *B. subtilis* YktC and YcsE functioned as 5′-nucleotidases, suggesting that they may represent two of the main enzymes involved in intracellular nucleoside/nucleotide metabolism. The other enzymes similar to *E. coli* NagD, which belong to the HADSF family (YutF, YsaA, YqeG, and AlaL), exhibited lower activity, although the data suggest they may function as minor 5′-nucleotidases. HADSF phosphatases often have broader substrate specificity and hydrolyze various phosphorylated compounds [5]. Thus, 5′-nucleotidases with lower substrate affinity might contribute to the balance of intracellular nucleotide concentrations as well as that of other phosphorylated metabolites. The *K*_M_ values of *E. coli* NagD for UMP, CMP, GMP, AMP, and G6P are 0.16 ± 0.038, 1.47 ± 0.044, 0.40 ± 0.130, 0.84 ± 0.250, and 5.90 ± 0.750 mM, respectively [4], which are smaller than those of YcsE and YktC (Tables 1 and 2, respectively). Therefore, *B. subtilis* YktC and YcsE might be less efficient as 5′-nucleotidase than NagD, but it is still suggestive that they may be able to function as two of the 5′-nucleotidases that are the first to respond to the accumulation of nucleotides, and the other enzymes may function when the concentration of nucleotides is in far excess.

Some of the genes encoding 5′-nucleotidases might be induced upon partial nucleotide limitation as previously reported [20]. Interestingly, the expression of *yqeG* is enhanced under oxidative stress, that of *ycsE* is induced by diamide or ethanol, and that of *yktC* is markedly enhanced in the presence of the various stressors, including diamide, ethanol, and high salt as well as at higher and lower temperatures [25]. These findings indicate that the intracellular levels of phosphorylated metabolites and nucleotides including 5′-nucleotides may be altered in the presence of these stressors, in particular oxidative stress. Our results show that inactivation of *yktC* led to increased sensitivity to diamide (Fig. 4). When the expression of *yqeG* was artificially modified, the cells grew normally in liquid medium and did not exhibit increased sensitivity to diamide, depending on repression of *yqeG* in the absence of IPTG (Fig. 4). Further, in the presence of diamide, the triple mutant NON06, lacking *ycsE* and *yktC* and with repressed *yqeG*, exhibited a growth defect similar to that of NON02 or NON03, which lacks *yktC* (Fig. 4). Therefore, we conclude that only YktC was involved in resistance to diamide-induced oxidative stress. However, further studies are required to elucidate how its enzymatic functions, including its 5′-nucleotidase activity, are related to resistance to the oxidative stress.

*B. subtilis* induces S-cysteinylation to protect protein thiols after exposure to diamide, because this bacterium does not produce thiols such as glutathione [35]. To our knowledge, enzymes with 5′-nucleotidase activity have not been demonstrated to be involved in resistance to oxidative stress. In response to oxidative stress, *Pseudomonas fluorescens* evokes a metabolic adaptation to increase NADPH synthesis and to decrease NADH production, accompanied by the induction and repression, respectively, of NAD^+^ kinase and NADP^+^ phosphatase, which regulate the levels of NAD^+^ and NADP^+^ [36]. Namely, oxidative stress represses the NADP^+^ phosphatase in *P. fluorescens* [36], whereas it induces *yktC* in *B. subtilis* [25]. If YktC might function as NADP^+^ phosphatase, it could decrease NADPH synthesis under oxidative stress, which is contradictory to the physiological demand. Therefore, YktC is unlikely to act as a NADP^+^ phosphatase, and we did not determine if NADP^+^ was a substrate of the phosphatase activity of YktC. On the other hand, *yqeG* is the first gene of a long operon comprising essential genes such as *nadD* that encodes nicotinamide-nucleotide adenylyltransferase required for *de novo* biosynthesis of NAD^+^ and NADP^+^ [37]. The induction of *yqeG* by oxidative stress, together with the other members of the operon, might be relevant to the enhanced NADPH synthesis as found in *P. fluorescens*. However, our results indicate that *yqeG* was dispensable, except for normal colony formation on solid medium (Fig. 4). We were therefore unable to determine the physiological function of *yqeG*.

In mammalian and plant cells, inositol monophosphatases play important roles in the metabolism of inositol polyphosphates and phosphatidylinositols [13, 38]. *De novo* phosphatidylinositol synthesis in mammals and *Saccharomyces cerevisia*e involves a reaction that combines CDP-diacylglycerol with *myo*-inositol. *myo*-Inositol is produced through the isomerization of glucose 6-phosphate to form MIMP [39] and the subsequent dephosphorylation of MIMP by inositol monophosphatase [16]. Further, MIMP is produced by the dephosphorylation of the second messenger inositol polyphosphates [40]. In *E. coli* and *B. subtilis*, however, phosphatidylinositols are not components of phospholipids usually present in the cell membrane [41, 42], and inositol 1-phosphate synthase has not been identified. Therefore, it is unlikely that MIMP occurs naturally in these bacteria and might serve as the physiological substrate of *E. coli* SuhB and *B. subtilis* YktC. In *E. coli*, a decrease in intracellular SuhB levels compensated the defects in *secY* and *rpoH* [43, 44]. The *secY* gene encodes a membrane component involved in protein secretion [45], and *rpoH* encodes σ^32^, a component of the RNA polymerase involved in heat-shock induction [46]. Therefore, defects in *secY* and *rpoH* impair secretion and the heat-shock response, respectively, which are involved in posttranslational quality control of proteins [18]. Therefore, SuhB may control the rates of peptide chain elongation and protein folding [42]. In contrast, the SuhB homolog of *P. aeruginosa* plays an important role in pathogenesis to control the genes required for acute and chronic infection [47]. *Burkholderia cepacia* SuhB is required for the secretion of proteins associated with motility and biofilm formation [48], suggesting that the physiological functions of SuhB homologs are complex, which may be true for *B. subtilis* YktC. Nevertheless, it is intriguing that MIMP is the best substrate for these enzymes. Kozloff and the colleagues detected a small amount of phosphatidylinositol from *E. coli* cells, implying the possibility of a weak phosphatidylinositol biosynthesis under some specific conditions [49]. *E. coli* SuhB and *B. subtilis* YktC might be involved in bacterial metabolism of phosphatidylinositol, which has not been characterized yet.

The broad substrate specificity of YktC revealed here implies the association of this enzyme with sugar-phosphate stress. Although sugars serve as energy and carbon sources, sugar phosphates are produced during their metabolism. Excess accumulation of sugar phosphates impairs cell growth [50] and may trigger cell death [51, 52] through an unknown mechanism. Similarly, accumulation of nucleotides is detrimental to the cell [34]. Therefore, by hydrolyzing sugar phosphates as well as nucleotides, YktC may play a role in correcting imbalances of metabolites. For example, we found that the *K*_M_ value of YktC for G6P was 2.6 ± 0.51 mM, which is greater than the reported intracellular concentrations of glucose 6-phosphate in *E. coli* (0.8–2.0 mM) [53]. However, under certain conditions, the intracellular concentration of G6P in *Lactococcus lactis* reaches 20–50 mM [54], indicating that G6P accumulates to higher levels in bacterial cells. Accordingly, in the presence of excess intracellular concentrations of G6P, YktC may hydrolyze it to intervene in glucose metabolism. This may be true as well for YcsE, because it hydrolyzed G6P in vitro more efficiently than YktC (Fig. 2). Similarly, YktC may hydrolyze β-glycerophosphate, which is a biosynthetic precursor of phospholipids [55], to interfere with the maintenance of the cell membrane. Therefore, their phosphatase activities with broad-specificity may exert pleiotropic effects on metabolism and other cellular functions. It would be worthwhile to perform metabolomic analyses on the mutants lacking *yktC* and *ycsE* to determine concentrations of the various metabolites, including 5′-nucleotides and other sugar phosphates, which would reinforce the hypothesis concerning the role of these enzymes.

Together with sodium glutamate, IMP and GMP are generally used as food additives to enhance taste. Industrial production of IMP is achieved using enzymatic conversion of inosine into IMP. *B. subtilis* strains producing large amounts of inosine in the fermentation medium were generated [56–60], and 5′-nucleotidase activity of these strains is remarkably increased, which may convert IMP into inosine [59]. Thus, optimizing the expression of genes encoding 5′-nucleotidases such as those studied here may further improve the fermentation of inosine production.

## Conclusions

*B. subtilis ycsE*, *araL*, *yutF*, *ysaA*, and *yqeG* show suggestive similarities to *E. coli nagD* encoding 5′-nucleotidase. Among the five, only YcsE exhibited significant 5′-nucleotidase activity with a broader specificity, whereas the other four enzymes had rather weak but suggestive activities with various capacities and substrate specificities. Interestingly, YqeG was required to maintain normal growth on solid medium. On the other hand, *B. subtilis yktC* shares high similarity with *E. coli suhB* encoding inositol monophosphatase, the gene product of which had 5′-nucleotidase activity preferential for GMP and IMP, and turned out to be involved in resistance to oxidative stress.

## Methods

### Bacterial strains, plasmids, oligonucleotide primers, and culture conditions

Bacterial strains and plasmids are listed in Table 3. Bacterial strains were usually grown aerobically at 37 °C and maintained in Luria-Bertani (LB) medium (Becton Dickinson, NJ, USA) containing 50 mg/l ampicillin and 50 mg/l kanamycin as required. Oligonucleotide primers are listed in Table 4. To assess the resistance to oxidative stress of *B. subtilis* strains, cells were inoculated into LB and allowed to grow. The oxidizing agent diamide was added to the exponentially growing culture at various concentrations, and growth was continuously monitored.

**Table 3:** Bacterial strains and plasmids

Strain and plasmid	Description	Source or reference
*B. subtilis*		
168	*trpC2*	Laboratory stock
NON01	*trpC2 ycsE*::*spc*	This study
NON02	*trpC2 yktC*::*cat*	This study
NON03	*trpC2 ycsE*::*spc yktC*::*cat*	This study
NON04	*trpC2 amyE*::(P*spac-yqeG erm*)	This study
NON05	*trpC2 amyE*::(P*spac-yqeG erm*) Δ*yqeG*::*kan*	This study
NON06	*trpC2 ycsE*::*spc yktC*::*cat amyE*::(P*spac-yqeG lacI erm*) Δ*yqeG*::*kan*	This study
TMO310	*trpC2 aprE*::(*spec lacI* P*spac-mazF*)	[62]
TMO311	*trpC2 aprE*::(*kan lacI* P*spac-mazF*)	[62]
*E. coli*		
DH5α	F^−^ Φ80*lacZ*ΔM15 Δ(*lacZYA*-*argF*) *U169 recA1 endA1 hsdR17*(*rK* ^−^, *mK* ^+^) *phoA supE44* λ^−^ *thi*-*1 gyrA96 relA1*	Laboratory stock
BL21(DE3)	F^−^ *ompT gal dcm lon hsdS*_*B*_(*r*_*B*_^−^, *m*_*B*_^−^) λ(DE3 [*lacI lacUV5*-*T7p07 ind1 sam7 nin5*]) [*malB* ^+^]_K-12_(λ^S^)	Laboratory stock
Plasmids		
pCRE-test	*cat*	[63]
pET28b	*kan*	Takara Bio
pET28b-araL	*kan, araL*	This work
pET28b-yutF	*kan, yutF*	This work
pET28b-yqeG	*kan, yqeG*	This work
pET28b-ysaA	*kan, ysaA*	This work
pET28b-ycsE	*kan, ycsE*	This work
pET28b-yktC	*kan, yktC*	This work
pMD20-araL	*amp*	This work
pMD20-yutF	*amp*	This work
pMD20-yqeG	*amp*	This work
pMD20-ysaA	*amp*	This work
pMD20-ycsE	*amp*	This work
pMD20-yktC	*amp*	This work
pMutin2	*erm lacI*	[64]

**Table 4:** Oligonucleotide primers

Name	Sequence (5′–3')^a^	Restriction site
araL-F	gggaattccatatgcgtattatggccagtcatgat	NdeI
araL-R	ccgctcgagtatcagaatcccctcctcagc	XhoI
yutF-F	cgcggatccatgaaaacatataaagggtattta	BamHI
yutF-R	cccaagcttaatgtatggaatccattcagt	HindIII
ysaA-F	cgcggatccatgaaagccgtattttttgattta	BamHI
ysaA-R	ccgctcgagtttctctaatataggaaacaa	XhoI
YqeG-F	gggaatccatatgttaaaaaagtttttt	NdeI
YqeG-R	cccaagcttttactcctcccactgaat	HindIII
ycsE-F	gggaattccatatgtctgtccaaagagaagatgta	NdeI
ycsE-R	ccgctcgagtagtacccaatggcgaatcgcttta	XhoI
yktC-F	cgcggatccatgacaaattggacggaaatcgat	BamHI
yktC-R	cccaagcttcttccgagcatgaagatactc	HindIII
ycsE-1	gtttattgatggcgtgac	
ycsE-2	ctgtgtagccttgagagtgatgaacagacatatatgtacctctc	
ycsE-3	tcactctcaaggctacacag	
ycsE-4	cgcaagcttcaaaaattatatggag	
ycsE-5	ctccatataatttttgaagcttgcggggtactataaaaaaagagagtcc	
ycsE-6	aacacttcatttgcggtc	
yktC-1	gttcgcctagagctgtaagcttc	
yktC −2	gccgatgataagctgtcaaagcaatctcatcgatttccgtcc	
yktC −3	tttgacagcttatcatcggc	
yktC −4	ttataaaagccagtcattaggc	
yktC −5	gcctaatgactggcttttataagtgctagccggaaacccatc	
yktC −6	gaacatctatcaccagacgacatc	
yqeG-d1	gacataatgcgccatttgatggttg	
yqeG-d2	gcttgagtcaattccgctgtcgctcaaacgaaaagggcacattcag	
yqeG-d3	cgacagcggaattgactcaagc	
yqeG-d4	cgcaagcttacgataaacccagc	
yqeG-d5	gctgggtttatcgtaagcttgcgcaaaacccgcacccttccc	
yqeG-d6	caggcagagcatatggatacg	
yqeG-s1	ccttccagggtatgtttctc	
yqeG-s2	gatactgcactatcaacacactctttcgacatggatgagcgatgatg	
yqeG-s3	aagagtgtgttgatagtgcagtatc	
yqeG-s4	caacaagctggggatccg	
yqeG-s5	cggatccccagcttgttgcgacaatcattttgaaagaaagaaaaaggg	
yqeG-s6	gatgacctcgtttccaccggcaaccttttccatttcttactcctcc	
yqeG-s7	ccggtggaaacgaggtcatc	
yqeG-s8	cacaaattaaaaactggtctgatcggcctaactcacattaattgcgttg	
yqeG-s9	cgatcagaccagtttttaatttgtg	
yqeG-s10	ttaacaaaattctccagtcttcacatcg	

^a^Restriction enzyme cleavage sites are underlined

### Construction of bacterial strains

*E. coli* strains expressing *B. subtilis* genes were constructed as follows: DNA fragments corresponding to the open reading frames of genes of interest were PCR-amplified using *B. subtilis* strain 168 chromosomal DNA (Table 3) as template and pairs of respective oligonucleotide primers with generation of unique restriction sites at their 5′-termini (araL-F/araL-R for *araL*, ycsE-F/ycsE-R for *ycsE*, yktC-F/yktC-R for *yktC*, yqeG-F/yqeG-R for *yqeG*, ysaA-F/ysaA-R for *ysaA*, and yutF-F/yutF-R for *yutF*) (Table 4). Each fragment was cloned into the pMD20-T plasmid using the Mighty TA-cloning kit (Takara Bio, Otsu, Japan) to yield pMD20-araL, pMD20-ycsE, pMD20-yktC, pMD20-yqeG, pMD20-ysaA, and pMD20-yutF, which were used to transform *E. coli* strain DH5α. After performing nucleotide sequencing to confirm the constructs, each recombinant plasmid was digested with the appropriate restriction enzyme(s) to prepare a DNA fragment corresponding to its respective open reading frame and then ligated to the arms of pET28b (Takara Bio) digested with the same enzyme(s). The plasmids pET28b-araL, pET28B-ycsE, pET28B-yktC, pET28B-yqeG, pET28B-ysaA, and pET28B-yutF were constructed to express each protein as a C-terminal His_6_-fusion, and expression was controlled by the T7 promoter in *E. coli* strain BL21(DE3) (Takara Bio).

Mutant strains of *B. subtilis* lacking *ycsE* and *yktC* were constructed using conventional marker replacement as follows (Additional file 1: Figure S1): To delete *ycsE*, two PCR fragments (each approximately 700-bp) corresponding to the upstream and downstream regions of *ycsE* were amplified from the DNA of *B. subtilis* strain 168 using the primer pairs ycsE-1/ycsE-2 and ycsE-5/ycsE-6, respectively (Table 4). A PCR fragment containing the spectinomycin-resistance gene cassette was amplified from the DNA of strain TMO310 (Table 3) using the primer pair ycsE-3/ycsE-4 (Table 4). The three PCR fragments were ligated using recombinant PCR with the primer pair ycsE-1/ycsE-6 to generate a single PCR fragment comprising the spectinomycin-resistance gene cassette flanked by the upstream and downstream regions of *ycsE. B. subtilis* strain 168 was transformed with the recombinant PCR fragment to confer resistance to spectinomycin, and a transformant with correct marker replacement was selected and designated strain NON01 (Table 3). To delete *yktC*, upstream and downstream regions of *yktC* were amplified using the primer pairs yktC-1/yktC-2 and yktC-5/yktC-6, respectively (Table 4). The chloramphenicol-resistance cassette was amplified from pCRE-test using the primer pair yktC-3/yktC-4 (Table 4). The three PCR fragments were ligated using recombinant PCR with the primer pair of yktC-1/yktC-6 to generate a single PCR fragment, which was integrated into chromosome of *B. subtilis* strain 168 to yield strain NON02 (Table 3). Strain NON01 was further transformed with NON02 DNA to confer resistance to chloramphenicol and generate strain NON03 lacking both *ycsE* and *yktC* (Table 3).

Another mutant that conditionally expressed *yqeG* was constructed as follows (Additional file 1: Figure S1). Five DNA fragments, corresponding to the C-terminal region of *amyE*, the stretch containing the erythromycin-resistance cassette and the *spac* promoter of pMutin2 (Table 3), the coding region of *yqeG*, the *lacI* gene of pMutin2, and the N-terminal region of *amyE*, were PCR-amplified using the primer pairs yqeG-s1/yqeG-s2, yqeG-s3/yqeG-s4, yqeG-s5/yqeG-s6, yqeG-s7/yqeG-s8, and yqeG-s9/yqeG-s10, respectively (Table 4). Using recombinant PCR with the primer pair yqeG-s1/yqeG-s10, all these five fragments were ligated in the order as described above and then integrated into the *amyE* locus of strain 168 to generate the erythromycin-resistant strain NON04 harboring an additional IPTG-inducible copy of *yqeG*.

Next, the endogenous *yqeG* gene of NON04 was deleted using marker replacement. Two DNA fragments comprising the upstream and downstream regions of *yqeG* (approximately 700 bp each) were amplified using the primer pairs yqeG-d1/yqeG-d2 and yqeG-d5/yqeG-d6, respectively (Table 4). The kanamycin-resistance cassette was amplified from the DNA of strain TMO311 using the primer pair yqeG-d3/yqeG-d4 (Table 4). Recombinant PCR was used to ligate together the three fragments using the primer pair of yqeG-d1/yqeG-d6t, and the ligation product was integrated into the chromosome of NON04 to yield strain NON05 in which expression of the additional *yqeG* was induced with 1 mM IPTG (Table 3). In NON05, *yqeG* was replaced with the kanamycin-resistance cassette so that the rest of operon comprising *yqeH*, *aroD*, *yqeI*, *nadD*, *yqeK*, *yqeL*, and *yqeM*, which reside downstream of *yqeG*, are constitutively expressed by read-through from the kanamycin-resistance gene. NON05 was transformed further with NON03 DNA to confer resistance to both chloramphenicol and spectinomycin inactivating *yktC* and *ycsE*, respectively (strain NON06).

Chromosomal DNAs of mutant strains of *B. subtilis* were subjected to diagnostic PCR experiments with the respective primer pairs used for recombinant PCR, and their correct construction was confirmed by appearance of the respective PCR fragments with altered length (Additional file 1: Figure S1).

### Purification of gene products produced in *E. coli*

*E. coli* strain BL21(DE3) bearing the pET28b derivatives were inoculated into 600 ml of LB medium containing kanamycin and were cultured at 37 °C with shaking at 180 rpm. When the optical density of the cultures reached 0.4–0.6, IPTG was added to a final concentration of 1 mM, the cells were cultivated further for 5 h, harvested using centrifugation, and resuspended into 10.8 ml of NP buffer (50 mM NaH_2_PO_4_, pH 8.0, and 300 mM NaCl). The suspension was chilled in an ice bath, mixed with 1.2 ml of 10 mg/ml lysozyme in NP buffer, incubated for 30 min, and sonicated to disrupt the cells. After centrifugation, the supernatant was subjected to His_6_-tag affinity purification using TALON Metal Affinity Resins (Takara Bio). The His_6_-tagged proteins were purified in a reaction buffer containing 1 mM NH_4_Cl, 250 mM KCl, 10 mM MgCl_2_, and 50 mM Tris–HCl (pH 7.8), and subjected to SDS-PAGE followed by Coomassie brilliant blue staining.

### Enzyme assay

The phosphatase assay was performed as previously described [61]. Each purified enzyme (0.25 mg/ml) was incubated with substrate in 50 μl of reaction buffer containing 1 mM NH_4_Cl, 250 mM KCl, 10 mM MgCl_2_, and 50 mM Tris–HCl (pH 7.8) at 37 °C, and the reaction was terminated using 50 μl of 10 % trichloroacetic acid. After centrifugation, the supernatant was mixed with 20 μl of solution A (10 % ascorbic acid in 2.25 M H_2_SO_4_) and 20 μl of solution B (2.4 % ammonium molybdate and 1 mg/ml potassium antimonyl tartrate in 2.25 M H_2_SO_4_). The mixture was incubated at room temperature for 10 min, and absorbance at 820 nm was measured to calculate the concentration of the released phosphate ion with reference to a standard curve made with various concentrations of inorganic phosphate. Trace of phosphate released in the reaction mixtures in the absence of purified enzyme was subtracted as background.

## Additional file

Additional file 1: Figure S1. Genetic organization of the altered chromosomal loci; Δ*ycsE*::*spc*(A), Δ*yktC*::*cat*(B), Δ*yqeG*::*kan*(C), and *amyE*::(P*spac*-*yqeG lacI erm*)(D). Genes and primers are indicated schematically by thick arrows and arrowheads, respectively. (PDF 144 kb)

## Abbreviations

G6P: Glucose 6-phosphate
HADSF: Haloalkane acid dehalogenase superfamily
IPTG: Isopropyl β-D-1-thiogalactopyranoside
MIMP: *myo*-Inositol 1-monophosphate
SDS-PAGE: Sodium dodecyl sulfate polyacrylamide gel electrophoresis
