# Supplementary material for: Bacillus subtilis 5′-nucleotidases with various functions and substrate specificities
Source: BMC Microbiol. 2016 Oct 26;16:249. doi: 10.1186/s12866-016-0866-5 (PMC5080769; doi:10.1186/s12866-016-0866-5)
Supplement: Additional file 1: Figure S1. — Genetic organization of the altered chromosomal loci; ΔycsE::spc(A), ΔyktC::cat(B), ΔyqeG::kan(C), and amyE::(Pspac-yqeG lacI erm)(D). Genes and primers are indicated schematically by thick arrows and arrowheads, respectively. (PDF 144 kb) [file 12866_2016_866_MOESM1_ESM.pdf]

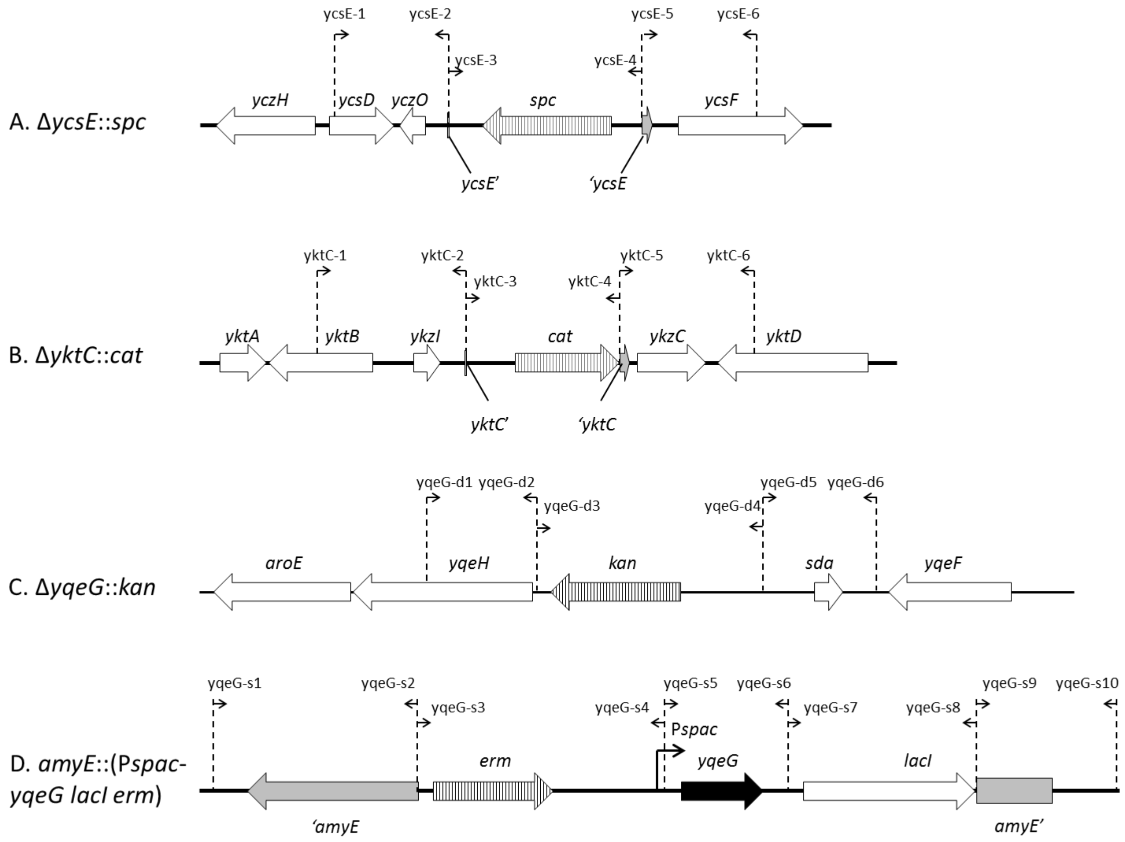

Fig. S1. Genetic organization of the altered chromosomal loci; Δ*ycsE*::*spc* (A), Δ*yktC*::*cat* (B), Δ*yqeG*::*kan* (C), and *amyE*::(P*spac*-*yqeG lacI erm*) (D). Genes and primers are indicated schematically by thick arrows and arrowheads, respectively.
